# Genomic epidemiology and heterogeneity of *Providencia* and their *bla*_NDM-1_-carrying plasmids

**DOI:** 10.1080/22221751.2023.2275596

**Published:** 2023-11-08

**Authors:** Peng Wang, Cuidan Li, Zhe Yin, Xiaoyuan Jiang, Xinyue Li, Xiaofei Mu, Nier Wu, Fei Chen, Dongsheng Zhou

**Affiliations:** a State Key Laboratory of Pathogen and Biosecurity, Beijing Institute of Microbiology and Epidemiology, Beijing, People’s Republic of China; b CAS Key Laboratory of Genome Sciences & Information, Beijing Institute of Genomics, Chinese Academy of Sciences and China National Center for Bioinformation, Beijing, People’s Republic of China; c University of Chinese Academy of Sciences, Beijing, People’s Republic of China; d State Key Laboratory of Pathogenesis, Prevention and Treatment of High Incidence Diseases in Central Asia, Urumqi, Xinjiang, People’s Republic of China

**Keywords:** *Providencia*, genome sequencing, plasmids, antimicrobial resistance, *bla*_NDM-1_

## Abstract

*Providencia* as an opportunistic pathogen can cause serious infection, and moreover the emergence of multi-drug-resistant *Providencia* strains poses a potentially life-threatening risk to public health. However, a comprehensive genomic study to reveal the population structure and dissemination of *Providencia* is still lacking. In this study, we conducted a genomic epidemiology analysis on the 580 global sequenced *Providencia* isolates, including 257 ones sequenced in this study (42 ones were fully sequenced). We established a genome sequence-based species classification scheme for *Providencia*, redefining the conventional 11 *Providencia* species into seven genocomplexes that were further divided into 18 genospecies, providing an extensively updated reference for *Providencia* species discrimination based on the largest *Providencia* genome dataset to date. We then dissected the profile of antimicrobial resistance genes and the prevalence of multi-drug-resistant *Providencia* strains among these genocomplexes/genospecies, disclosing the presence of diverse and abundant antimicrobial resistance genes and high resistance ratios against multiple classes of drugs in *Providencia*. We further dissected the genetic basis for the spread of *bla*_NDM-1_ in *Providencia*. *bla*_NDM-1_ genes were mainly carried by five incompatible (Inc) groups of plasmids: IncC, IncW, Inc_pPROV114-NR_, Inc_pCHS4.1-3_, and Inc_pPrY2001_, and the last three were newly designated in this study. By tracking the spread of *bla*_NDM-1_-carrying plasmids, IncC, Inc_pPROV114-NR_, Inc_pCHS4.1-3_, and Inc_pPrY2001_ plasmids were found to be highly involved in parallel horizontal transfer or vertical clonal expansion of *bla*_NDM-1_ among *Providencia*. Overall, our study provided a comprehensive genomic view of species differentiation, antimicrobial resistance prevalence, and plasmid-mediated *bla*_NDM-1_ dissemination in *Providencia*.

## Introduction

*Providencia* is a genus of Gram-negative, motile bacteria in the family *Morganellaceae*, and *Providencia* strains are commonly found in water, soil, and animal reservoirs [1]. *Providencia* can act as an opportunistic pathogen causing nosocomial [2] or foodborne [3] infections. This genus majorly contains 11 species including *P. alcalifaciens* [3], *P. burhodogranaeriae* [4], *P. heimbachae* [5], *P. huaxiensis* [6], *P. rettgeri* [7], *P. rustigianii* [8], *P. sneebia* [4], *P. stuartii* [9], *P. thailandensis* [10], *P. vermicola* [11], and *P. friedericiana* [12] (a heterotypic synonym of *P. rustigianii* [8]). It has been reported that strains within a specific *Providencia* species exhibit significant genomic diversity [13]. However, there is still a lack of comprehensive and large-scale genomic investigation to reveal the genetic heterogeneity in *Providencia*.

Historically, the identification of certain *Providencia* species primarily relied on distinct biochemical reactions. These species are characterized by a positive reaction to phenylalanine deaminase and a negative reaction to lysine, ornithine decarboxylase, and arginine dihydrolase. Notably, the production of acid from d-mannose serves as an indicative trait. Within this context, *P. rettgeri* and *P. stuartii* are typically differentiated by their respective abilities to produce acid from trehalose and d-arabitol, with *P. rettgeri* uniquely producing acid from erythritol. However, for several conventional species, specific biochemical reactions are absent [14]. At present, advancements in sequencing technology have promoted the use of 16S rDNA for identifying *Providencia* isolates, achieved through PCR amplification. However, this method has limitations, with high sequence similarities often resulting in misclassifications. Thus, a large-scale genomic investigation is important for the precise identification and classification of *Providencia* species, ensuring the accurate differentiation and understanding of each species within the context of their genomic profiles.

*Providencia* species are known to exhibit multi-drug resistance (MDR) due to their intrinsic resistance to penicillins and the first-generation cephalosporins [14], aminoglycosides [15], tetracyclines [16], and polymyxin [17]. Moreover, extensive use and frequent misuse of antibiotics have led to the emergence of extended-spectrum β-lactamase- or even carbapenemase-producing *Providencia* strains, exacerbating the MDR problem in *Providencia* [18,19]. It has been revealed that the frequent emergence of carbapenemase-producing *Providencia* strains is mainly due to the spread of *bla*_NDM-1_ gene [2,20]. IncC plasmids that carry *bla*_NDM-1_ have been widely detected in *P. stuartii* from distrust Romanian [21] and Italy hospitals [22], and several additional Inc (incompatible) groups of *bla*_NDM-1_-harbouring plasmids have been identified in *Providencia* [18,23–26], indicating diverse types of plasmids contribute to the spread of *bla*_NDM-1_ in *Providencia*. However, a genomic view of the dissemination of *bla*_NDM-1_-carrying plasmids among a large collection of *Providencia* strains is lacking.

In this study, we performed genome sequencing of 257 Chinese *Providencia* isolates (42 of them obtained complete genome sequences). Together with the 323 *Providencia* genomes available in GenBank, we used a total collection of the 580 global *Providencia* sequenced isolated for further genomic epidemiology analyses, aiming to establish a genome sequence-based classification scheme, disclose the prevalence of antimicrobial resistance and dissect the plasmid-mediated *bla*_NDM-1_ dissemination in *Providencia*.

## Materials and methods

### Bacterial isolates and identification

From 2010 to 2018, 22.798 bacteria were collected in over 200 hospitals in China by our group, most of which were identified as *Klebsiella* (31.31%), *Escherichia coli* (21.34%), *Pseudomonas* (14.51%), *Acinetobacter* (10.13%). Among these 22,798 isolates, 303 isolates were initially identified as *Providencia* using Vitek or mass spectrometry in hospitals. After eliminating 6 unsuccessfully cultured isolates and 27 ones without the *Providencia* 16S rDNA gene, 270 isolates were obtained. Bacterial genomic DNAs were then extracted using a Qiagen UltraClean Microbial DNA Isolation Kit, followed by sequencing by Illumina platform. 13 isolates with poorly sequencing quality were subsequently excluded. 257 *Providencia* isolates were finally enrolled in this study between 2010 and 2018, whose species were further identified by ANI analyzing their whole genome information (Figure S1).

### Bacterial phenotypic resistance assay

The bacterial antimicrobial susceptibility was tested using BioMérieux VITEK 2 and interpreted as per the 2020 Clinical and Laboratory Standards Institute (CLSI) guidelines [27]. The activity of class A/B/D carbapenemases in bacterial cell extracts was detected by a modified CarbaNP test [28].

### Genomic DNA sequencing, sequence assembly, and annotation

All the 257 Chinese *Providencia* isolates were subjected to draft-genome sequencing using a paired-end library with an average insert size of 350 bp (ranging from 150 bp to 600 bp) on a HiSeq sequencer (Illumina, CA, USA). In addition, 42 (Table S1) of them were subjected to complete genome sequencing with a sheared DNA library with an average size of 15 kb (ranging from 10 kb to 20 kb) on a PacBio RSII sequencer (Pacific Biosciences, CA, USA). The quality control analysis of sequencing data was conducted using NanoPack [29] and FastQC (https://www.bioinformatics.babraham.ac.uk/projects/fastqc/). Sequence assembly and annotation were performed as previously described [30–32].

### Phylogenomic analysis and average nucleotide identity (ANI) analysis

The pairwise ANI values of the 580 global *Providencia* genomes were calculated using FastANI [33], and the ANI heatmap was generated by the python seaborn package. The *Providencia* genome sequences were aligned to the complete chromosome sequence (GenBank accession number CP029736) of *P. rettgeri* AR_0082, and the core single nucleotide polymorphisms (SNPs) were identified by Mummer v3.2 (https://mummer.sourceforge.net/). All the SNPs in the repetitive DNA regions were identified and filtered by RepeatMasker (http://www.repeatmasker.org/). Based on the final core SNPs, the maximum-likelihood phylogenetic tree was constructed using RAxML [34] under the GTR model with a bootstrap iteration of 1000 and visualized using iTOL (https://itol.embl.de).

### Plasmid analysis

All the fully sequenced *bla*_NDM-1_-carrying plasmids from GenBank (last accessed Dec 20, 2021) together with those determined in this study were used as the reference to assemble and align the draft sequences of the rest *bla*_NDM-1_-carrying plasmids in the 580 global *Providencia* genomes using BLAST [35] and custom Perl scripts. The five Inc groups and core backbone *rep* (replication) and *par* (partition) genes were determined for all the *bla*_NDM-1_-carrying plasmids in the 580 global *Providencia* genomes. To achieve high accuracy, the assembled draft plasmid sequences met the following three criteria [36,37]: the *bla*_NDM-1_-embedded contigs had 100% query coverage and ≥99% identity with corresponding reference plasmids; the *bla*_NDM-1_-carrying contigs and the *rep*-carrying contigs of the same plasmid had similar sequencing depths; each draft plasmid sequence had ≥70% query coverage and ≥94% identity with the corresponding reference plasmids. The alignment rings of the five Inc groups were visualized by BRIG [38].

### Statistical analysis

Statistical analyses were preformed using R package v.3.271 (http://www.r-project.org). Chi-squared test or Fisher's exact test was used to determine the significant relationships between categorical variables. Wilcoxon test, a non-parametric test for the comparison of two groups, was adopted to determine the significant differences for the medians of two independent samples.

### Data availability

The complete chromosome and plasmid sequences of the fully sequenced 42 isolates (including 33 *bla*_NDM-1_-positive isolates and three *bla*_VIM-4_-positive isolates) were submitted to GenBank with the accession numbers listed in Table S1. The 257-genome sequencing data have been uploaded to the SRA database under BioProject PRJNA1013098. The assembled genome sequences of the isolates were submitted to GenBank under BioProject PRJNA828374.

## Results

### Genome sequence-based classification of *Providencia* into diversified genocomplexes and genospecies

**Figure 1.**
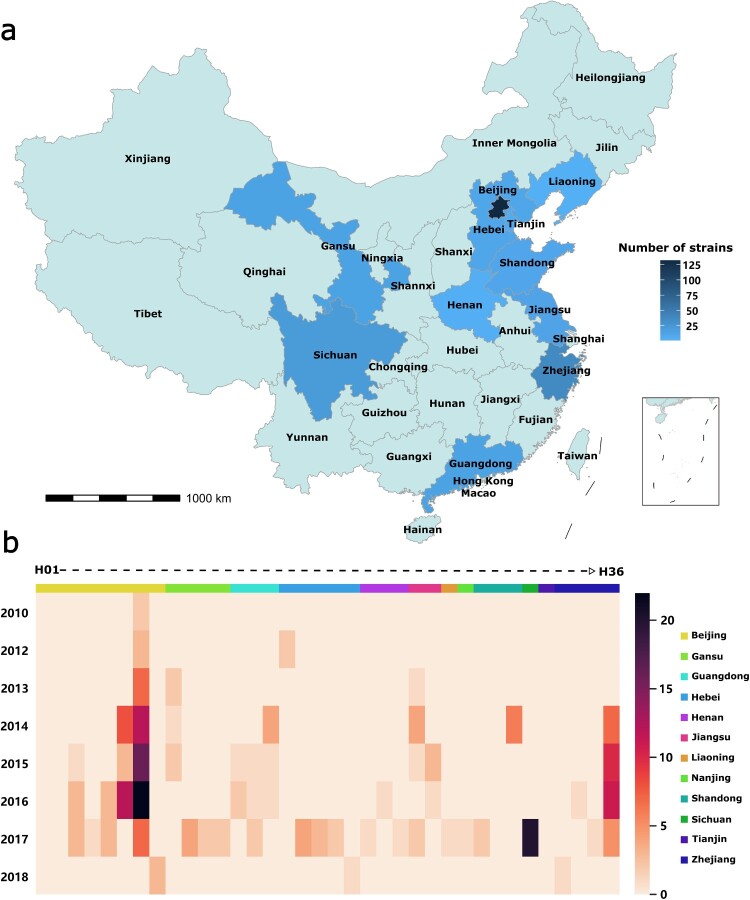
Spatial-temporal distribution of our 257 Chinese *Providencia* isolates. (a) Distribution of the 257 isolates among 12 provinces of China. (b) Distribution of the 257 isolates among different years, provinces, and hospitals. The 37 hospitals (designated H1 to H36) were assigned to the 12 provinces with different colours.

**Figure 2.**
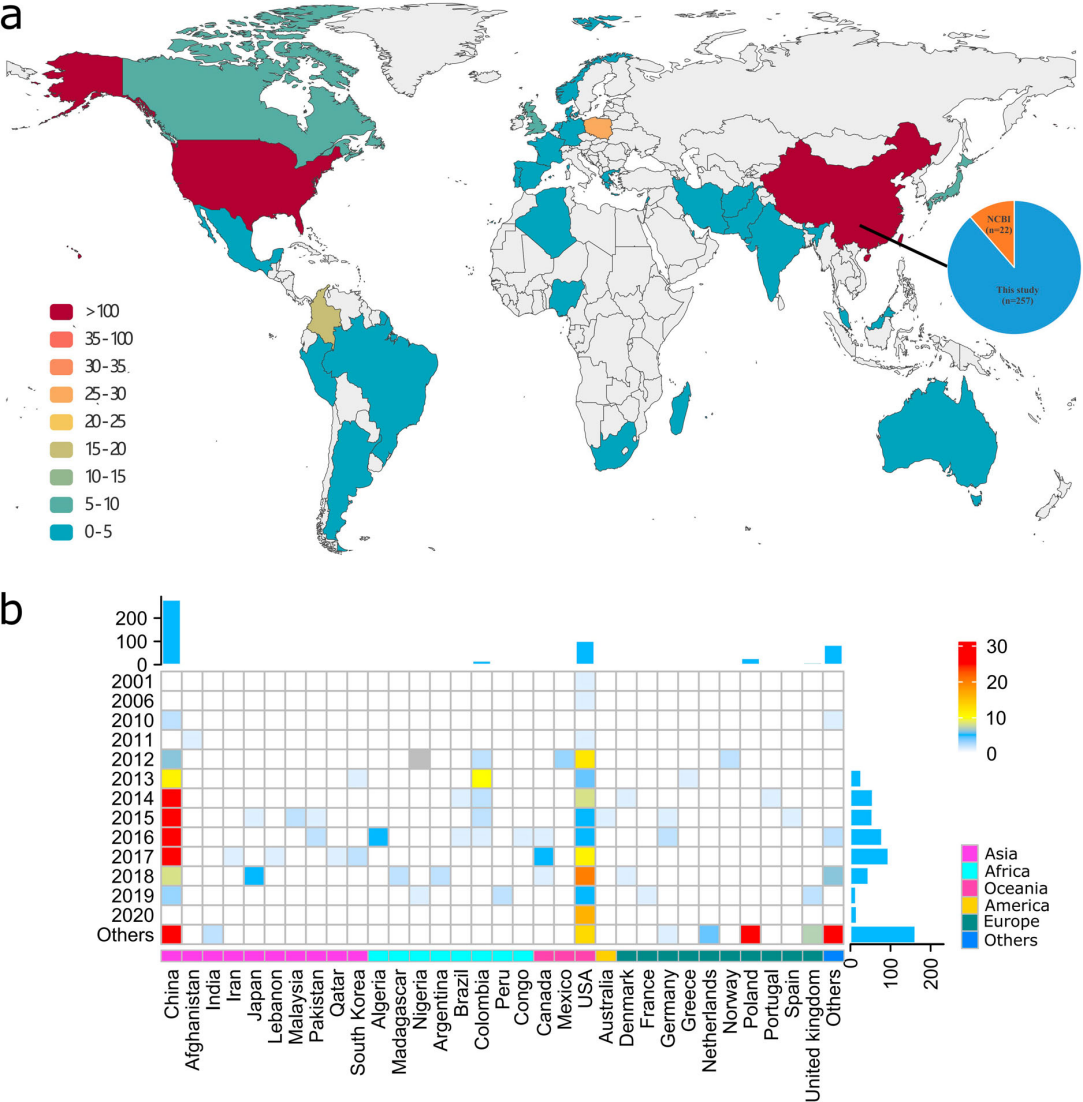
Spatial-temporal distribution of the 580 global *Providencia* isolates. (a) Spatial distribution of the 580 global *Providencia* isolates. Different colours represent the number of isolates. The pie chart shows the percentages of our 257 Chinese *Providencia* isolates and additional 22 Chinese isolates from GenBank. (b) Distribution of the 580 global *Providencia* isolates in different years and countries. The heatmap displays the number of isolates in each year and country. The total numbers of isolates collected in a year or in a country were shown in the bar charts on the right or top of the figure, respectively. The countries located on the same continent were marked in the same colour at the bottom of the figure.

The 257 Chinese *Providencia* isolates collected in this study (Figure 1 and Table S1) were determined with draft-genome sequences, among which 42 ones (including 33 *bla*_NDM-1_-positive ones) were further determined with complete genome sequences. Together with the 323 *Providencia* genomes available in GenBank as of February 1st, 2022, a total collection of 580 global *Providencia* genomes from 32 countries were applied for further analysis, covering all the 11 conventional *Providencia* species (Figure 2). These 580 isolates include 328 *P. rettgeri* isolates, 137 *P. stuartii* isolates, 73 *P. alcalifaciens* isolates, 16 *P. rustigianii* isolates, eight *P. heimbachae* isolates, four *P. vermicola* isolates, three *P. huaxiensis* isolates, one *P. sneebi* isolate, one *P. burhodogranaeriae* isolate, one *P. thailandensis* isolate, and one *P. friedericiana* isolate. There were seven additional *Providencia* isolates that could not be classified into any of these conventional species due to their low genetic similarity to these species.

**Figure 3.**
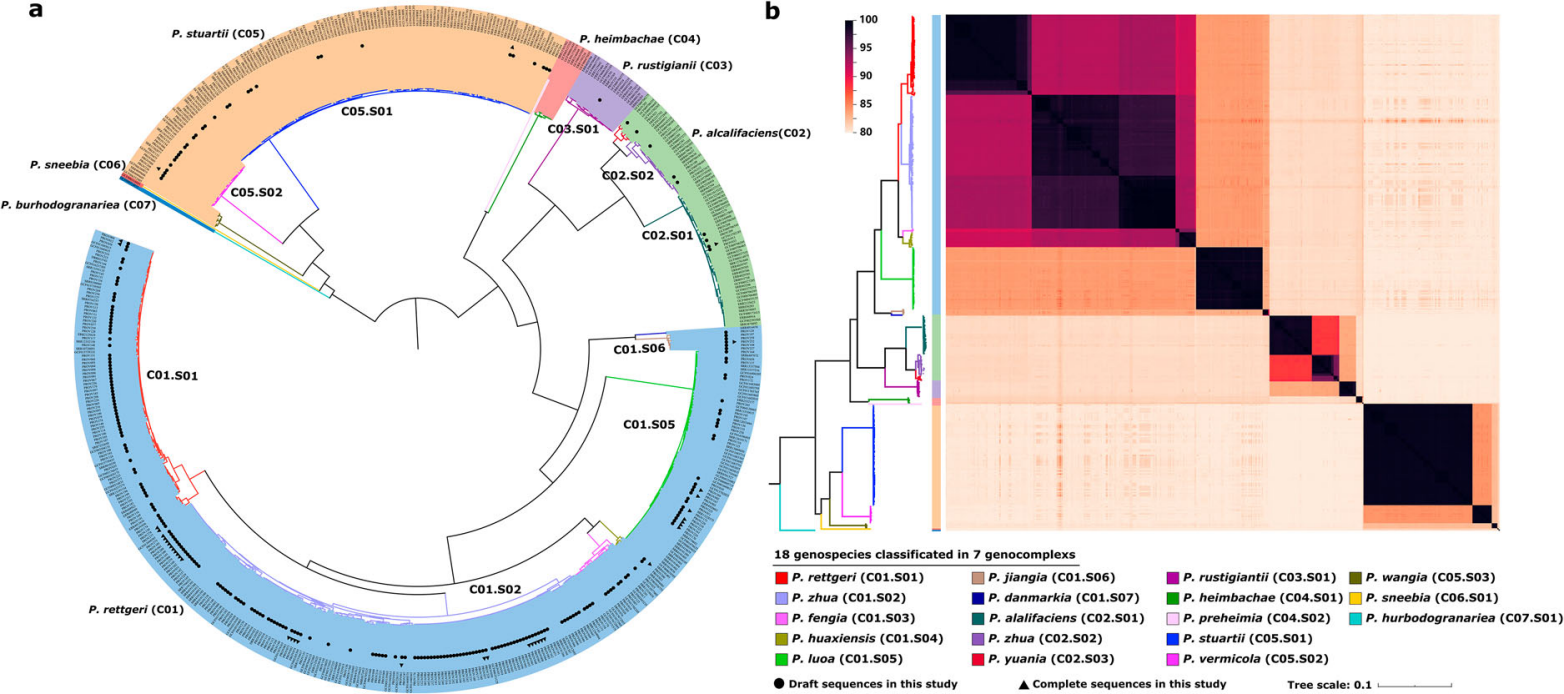
Genome sequence-based classification of the 580 global *Providencia* isolates. (a) A maximum-likelihood phylogenetic tree based on the 13,499 core SNPs from the 580 genomes. *Providencia* AR_0082 was used as the reference chromosome for SNP calling. The confidence value in the major branch nodes were over 95%. The conventional classification was marked on the circle of the phylogenetic tree, and the typical reference isolates of the 11 conventional *Providencia* species was marked in asterisks with different colours. Seven primary phylogroups (i.e. genocomplexes) with significant phylogenetic distance were distinguished in different colours. (b) A heatmap of pairwise ANI of the 580 isolates. The pairwise ANI values of the 580 isolates range from 79.73% to 99.99%. The phylogenetic trees were used as the references and placed on the upper and right sides of the ANI heatmap. The order of isolates on the heatmap was corresponding to the isolate position on the phylogenetic tree. The genocomplexes and genospecies were cross-determined according to the topology of the phylogenetic tree and the ANI heatmap.

To perform phylogenetic analysis, a maximum-likelihood phylogenetic tree was constructed based on the 13,499 core SNPs from these 580 *Providencia* genomes (Figure 3(a)). The resulting phylogenetic topology revealed seven primary genocomplexes, including *P. rettgeri* (C01), *P. alcalifaciens* (C02), *P. rustigianii* (C03), *P. heimbachae* (C04), *P. stuartii* (C05), *P. sneebia* (C06), and *P. burhodogranariea* (C07). These genocomplexes could be further classified into 18 genospecies, nine of which were newly reported (Table 1). These phylogenetic classification results were verified by ANI values (Figure 2(b)), with ANI greater than 98.5% among the same genospecies, greater than 82% among the same genocomplexes, and less than 82% between different genocomplexes. Herein, the *P. rettgeri* (C01) genocomplex (*n* = 338/580) was found to be highly dominant in *Providencia*, and it was further classified into seven genospecies: *P. rettgeri* (C01.S01), *P. zhua* (C01.S02), *P. fengia* (C01.S03), *P. huaxiensis* (C01.S04), *P. luoa* (C01.S05), *P. jiangia* (C01.S06), and *P. danmarkia* (C01.S07).

**Table 1.** Designation of genocomplexes and genospecies in *Providencia*.

Genocomplexes		Genospecies				
Designation	ID	Designation	ID	Reference strain	Accession number	Reference
*P. rettgeri*	C01	*P. rettgeri*	S01	ATCC 29944/NCTC 11801	GCF_003204135^$^	[7]
		P. zhua*	S02	PROV006^#^	CP096367^$^	This Study
		P. fengi*	S03	PROV200^#^	CP096307^$^	This study
		*P. huaxiensis*	S04	KCTC 62577	GCF_002843235^$^	[6]
		P. luoa*	S05	PROV222^#^	CP096304^$^	This study
		P. jiangia*	S06	PROV252^#^	CP096294^$^	This study
		P. danmarkia*	S07	1D-1086	ERR4014438	-
*P. alcalifaciens*	C02	*P. alcalifaciens*	S01	ATCC 9886	GCF_002393505^$^	[9]
		P. zhoua*	S02	PROV188^#^	CP097291^$^	This study
		P. yuania*	S03	PROV023^#^	CP059348^$^	This study
*P. rustigianii*	C03	*P. rustigianii*	S01	ATCC 33673	GCF_000156395^$^	[8]
*P. heimbachae*	C04	*P. heimbachae*	S01	ATCC 35613	GCF_900475855^$^	[5]
		P. preheimia*	S02	bc_500_040518_04	SRR12761562	-
*P. stuartii*	C05	*P. stuartii*	S01	ATCC 29914/KCTC 2568	GCF_010669105^$^	[10]
		*P. vermicola*	S02	DSM 17385	GCF_010748935^$^	[11]
		P. wangia*	S03	PROV236^#^	CP096297^$^	This study
*P. sneebia*	C06	P. sneebia*	S01	ATCC BAA-1589	GCF_000314895^$^	[4]
*P. burhodogranariea*	C07	*P. burhodogranariea*	S01	ATCC BAA-1590	GCF_000314855^$^	[4]

* Designated in this study.

^#^ Collected and fully sequenced in this study.

^$^ Complete genome sequence.

**Figure 4.**
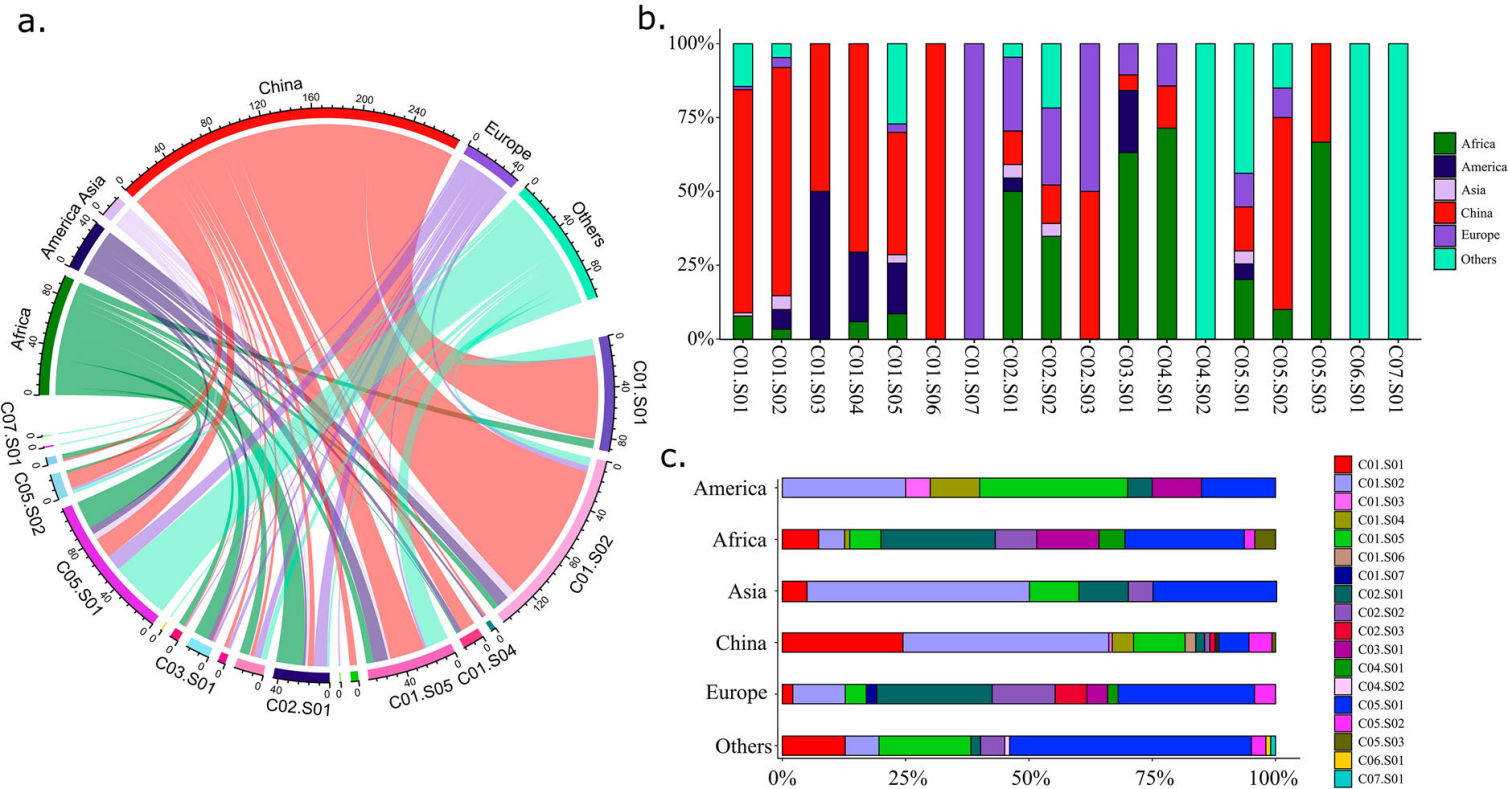
Prevalence of the 580 global *Providencia* isolates in different genospecies. (a) The prevalence of isolates in the major geographic regions. The links on the circle represented the composition of the isolates according to geographic region or genospecies. The edge size on the circle corresponds to the number of isolates. (b) The percentage of isolates collected from different geographic regions in each genospecies. The numbers on the *y*-axis represent the percentage values of the isolates from different geographic regions. (c) The percentage of isolates from different genospecies in each geographic region. The numbers on the *x*-axis represent the percentage values.

The 279 Chinese *Providencia* isolates (including 257 collected in this study, and additional 22 from GenBank) could be assigned into four genocomplexes and 13 genospecies, and most (214/279, 76.70%) of them belonged to the three genospecies C01.S01 (68/279, 24.37%), C01.S02 (116/279, 41.58%), and C01.S05 (30/279, 10.75%) (Figure 3). Therefore, C01.S01, C01.S02, and C01.S05 represented the three dominant *Providencia* genospecies in China. The above three genospecies together with C05.S01 were found to be principally prevalent in America, Africa, and Europe regions (Figure 4).

### Broad-spectrum antimicrobial resistance phenotype in *Providencia* from China

Drug susceptibility testing of our 257 Chinese *Providencia* isolates was performed for five categories (β-lactams, aminoglycosides, quinolones, furanea, and sulphanilamides) of 20 different antibiotics (Figure S4): these isolates showed almost full (>93.38%) resistance for four antibiotics (ampicillin, ampicillin/sulbactam, cefazolin, and furadantin) and >50% resistance ratios for five antibiotics (cefuroxime axetil, cefuroxime, ciprofloxacin, levofloxacin, and trimethoprim), followed by the six remaining antibiotics (piperacillin, ceftazidime, ceftriaxone, cefepime, gentamicin, and nebcin) with moderate resistance ratios ranged from 20% to 50%. In particular, our 257 Chinese *Providencia* isolates displayed relatively low resistance ratios (<20%) for five antibiotics, including piperacillin/tazobactam (18.29%, 47/257), cefotetan (14.40%, 37/257), aztreonam (17.51%, 45/257), meropenem (14.40%, 37/257), and amikacin (12.84%, 33/257), indicating that any of these five antibiotics could be the first choice for the treatment of *Providencia* infection in China.

The drug-resistance profiles of different genocomplexes/genospecies were shown in Figure S5. All the *Providencia* genocomplexes/genospecies exhibited the intrinsic resistance against ampicillin, ampicillin/sulbactam, cefazolin, and furadantin. Among them, the C01 (*P. rettgeri*) isolates presented more than 50% resistance ratios for at least 10 antibiotics. Compared to the other genocomplexes, C01 exhibited much higher resistance to ceftazidime (*P*-value = 0.006048), ceftriaxone (*P*-value = 0.000501), cefepime (*P*-value = 1.7e-08), meropenem (*P*-value = 0.00226), ciprofloxacin (*P*-value = 0.003564), and levofloxacin (*P*-value = 7.579e-05). Overall, the above findings confirmed the existence of severe antimicrobial resistance phenotype in *Providencia* from China.

### Abundant antimicrobial resistance genes in *Providencia*

**Figure 5.**
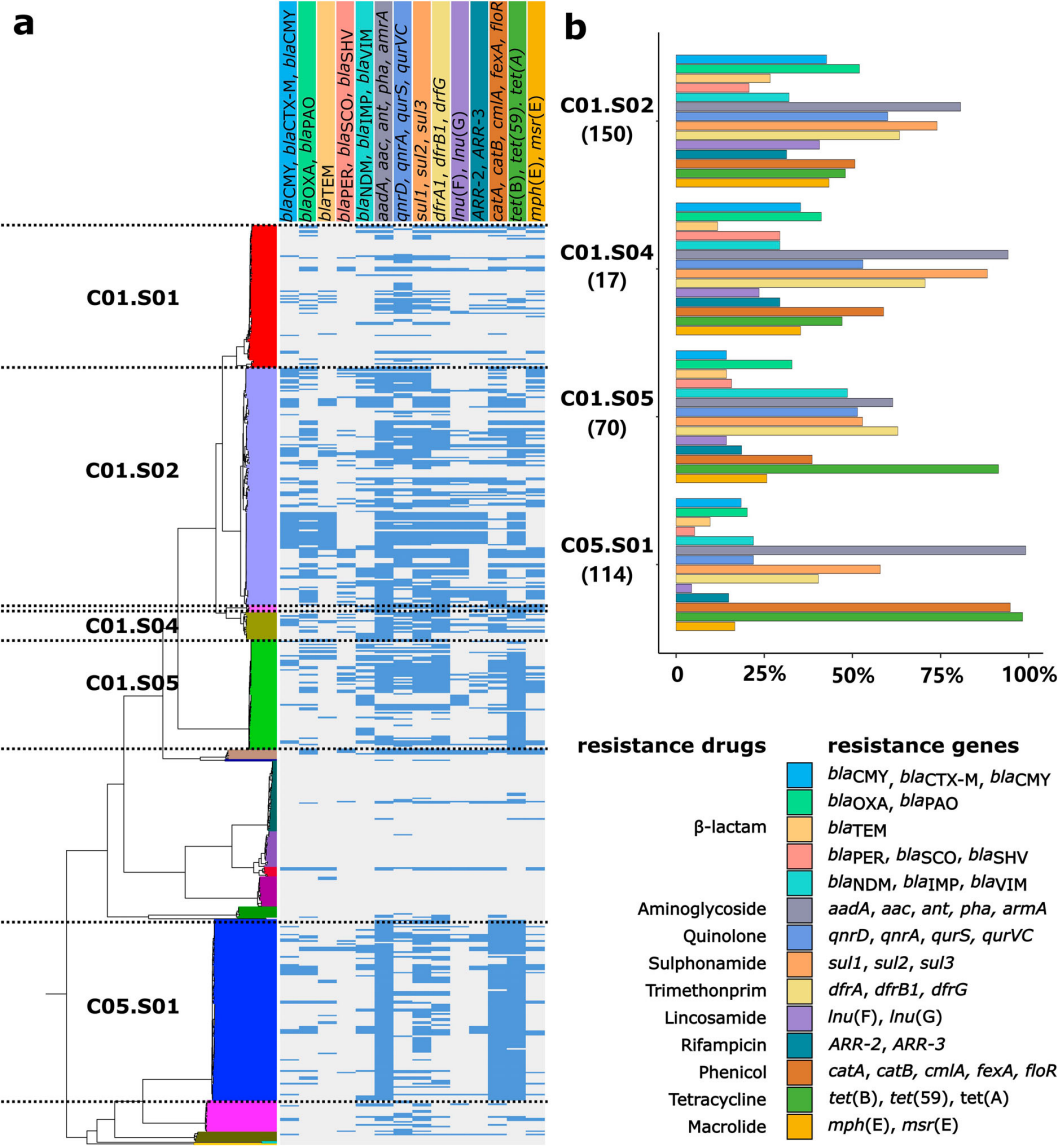
Prevalence of antimicrobial resistance genes in the 580 global *Providencia* isolates. (a) Shown was the rectangular format of the core SNP-based phylogenetic tree in Figure 3. The antimicrobial resistance genes harboured in the isolates were marked with blue blocks in the columns. (b) Bar charts showed the detection ratios of antimicrobial resistance genes in different genospecies. The corresponding relationships between drugs and resistance genes were shown.

We identified 173 different antimicrobial resistance genes belonging to 16 categories within the 580 global *Providencia* genomes (Figure S2). Our results showed serious antimicrobial resistance of *Providencia* with 10 categories of resistance genes having ratios over 15% in the following ranking order (from high to low) (Figure 5(a)): aminoglycoside-resistance genes (348/580, 60%), tetracycline-resistance genes (288/580, 49.65%), sulphonamide-resistance genes (283/580, 48.79%), β-lactam-resistance genes (282/580, 48.62%), phenicol-resistance genes (256/580, 44.14%), trimethoprim-resistance genes (236/580, 40.69%), quinolone-resistance genes (219/580, 37.76%), macrolide-resistance genes (143/580, 24.66%), rifampicin-resistance genes (106/580, 18.2%), and lincosamide-resistance genes (100/580, 17.24%).

We further analysed the prevalence of these resistance genes among the genocomplexes/genospecies. The results showed that these resistance genes were mainly detected in the *P. rettgeri* (C01) and *P. stuartii* (C05) genocomplexes, with six categories of resistance genes having ratios over 50%, particularly in the C01.S02, C01.S04, C01.S05, and C05.S01 genospecies (Figure 5(b)). C01.S02 contained the largest categories (10/16) of resistance genes with detection ratios over 50%; C01.S05 had the highest detection ratio (34/70, 48.5%) of carbapenemase genes *bla*_NDM_, *bla*_IMP_, and *bla*_VIM_; in C05.S01, the aminoglycoside-resistance gene *aac(2′)-la* (113/114, 99.12%), the phenicol-resistance gene *catA3* (107/114, 93.86%), and the tetracycline-resistance gene *tet(B)* (112/114, 98.25%) were identified in almost all the genomes, indicating a stable inherited behaviour (Figure S2).

Compared to the *Providencia* isolates from other countries, the Chinese isolates presented significantly higher resistance gene ratios in C01.S01 (*P*-value = 0.0041) and C01.S02 (*P*-value = 0.0015) (Figure S3), indicating different resistance-gene prevalence profiles between China and other countries [22,39,40]. *Providencia* strains harboured abundant antimicrobial resistance genes and might serve as reservoirs for resistance genes, exacerbating the spread of resistance genes among different bacteria.

Two carbapenemase genes *bla*_NDM-1_ (33/257, 12.84%) and *bla*_IMP_ (3/257, 1.17%) were identified in our 257 Chinese *Providencia* isolates, and their class B carbapenemase activities were further validated by CarbaNP and meropenem drug susceptibility tests (Table S1). In addition, three carbapenemase genes *bla*_NDM-1_ (82/580, 14.13%), *bla*_IMP_ (38/580, 6.55%), and *bla*_VIM_ (7/580, 1.21%) were identified in the 580 global *Providencia* genomes (Figure S2). Carbapenemase genes were detected in only C01 from China, while they were detected in C01 and C05 from other countries (Figure S3) [18,21,26,41]. Overall, carbapenemase genes were detected at relatively low frequency and *bla*_NDM-1_ represented the major disseminated carbapenemase gene in *Providencia*.

### Plasmid-mediated dissemination of *bla*_NDM-1_ in *Providencia*

To exactly analyse the mobile genetic platforms for *bla*_NDM-1_, the complete genome sequences of all the 33 *bla*_NDM-1_-positive isolates from our 257 Chinese *Providencia* isolates were obtained through second- and third-generation sequencing methods (Table S1). Plasmid-borne *bla*_NDM-1_ genes were observed in 31 (93.94%) of these 33 isolates (Table S1), while chromosome-borne *bla*_NDM-1_ genes were identified in the rest two isolates and moreover these two *bla*_NDM-1_ genes together with a wealth of additional resistance genes were embedded within huge and complex accessory genetic elements acquired by the two chromosomes [42]. The 31 *bla*_NDM-1_-harbouring plasmids could be classified into five Inc groups, namely IncC (16/31, 51.61%), Inc_pCHS4.1-3_ (6/31, 19.35%), Inc_pPrY2001_ (4/31, 12.90%), Inc_pPROV114-NR_ (4/31, 12.90%), and IncW (1/31, 3.23%) (Table S1). Similarly, *bla*_NDM-1_-carrying plasmids of the above five Inc groups were found in 72 (87.80%) of the 82 *bla*_NDM-1_-positive ones from the global 580 genomes, including IncC (36/72, 50%), Inc_pPrY2001_ (20/72, 27.78%), Inc_pCHS4.1-3_ (10/72, 13.89%), Inc_pPROV114-NR_ (5/72, 6.94%), and IncW (1/72, 1.39%) (Table S2). Inc_pPrY2001_, Inc_pCHS4.1-3_, and Inc_pPROV114-NR_ were newly designed in this study because the corresponding plasmids had novel replicons and their core backbone regions had low homology with existing Inc groups of plasmids. The *bla*_NDM-1_-containing plasmids in each Inc group carried the identical core backbone *rep* and *par* genes and shared the large conserved backbone regions, and *bla*_NDM-1_-surrounding regions were recognized as the accessory components of the corresponding plasmids (Figure S6).

**Figure 6.**
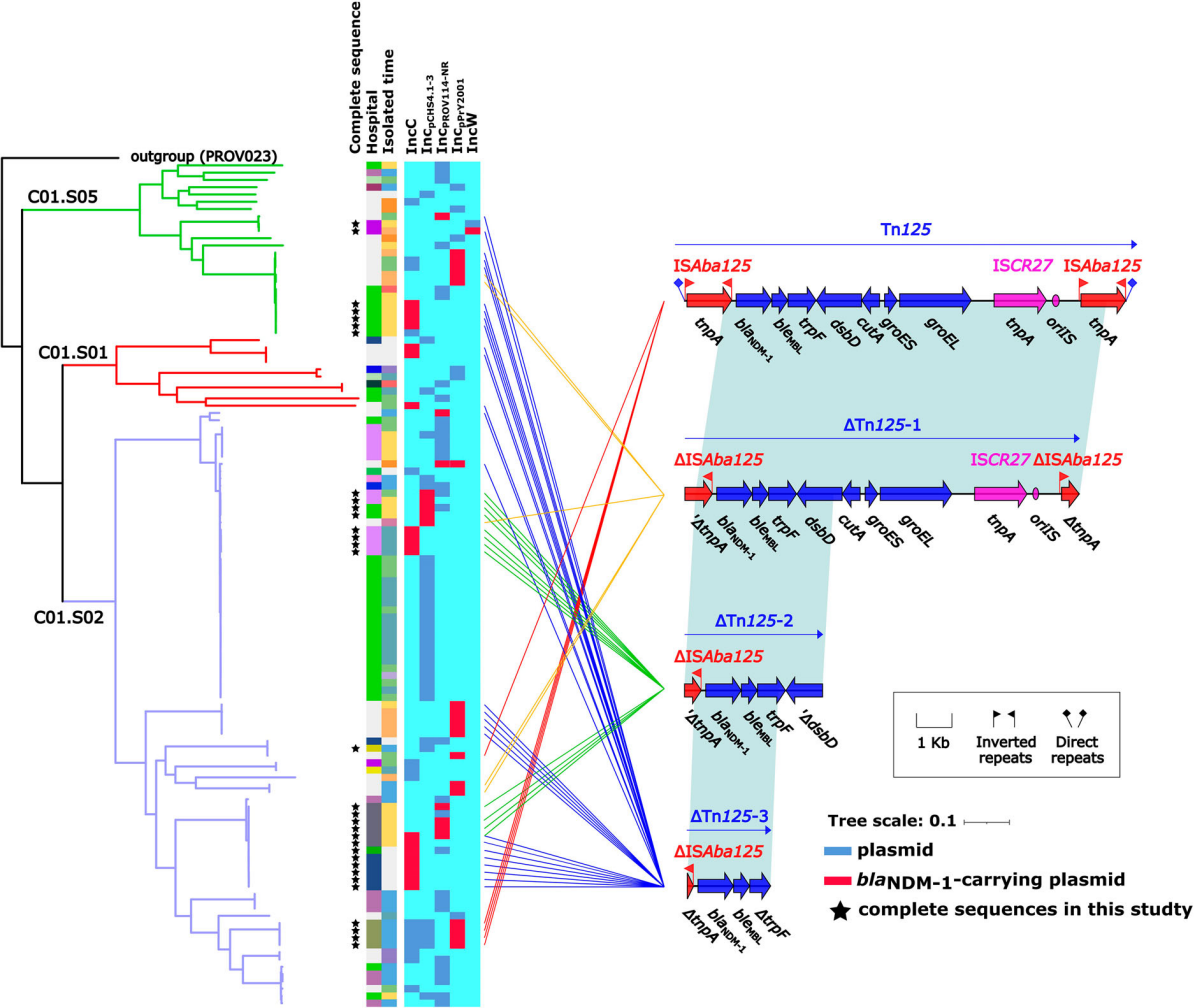
Dissemination of *bla*_NDM-1_-carrying plasmids in genospecies C01.S01, C01.S02, and C01.S05. A recombination-free core SNP-based phylogenetic tree was constructed using a collection of 116 *Providencia* isolates, which harboured IncC, Inc_pCHS4.1-3_, Inc_pPrY2001_, Inc_pPROV114-NR_, and IncW plasmids (either carrying *bla*_NDM-1_ or not), from the three dominant genospecies C01.S01, C01.S02, and C01.S05. All the 116 genomes were aligned to the complete chromosome sequence of *P. rettgeri* AR_0082, and the 168,349 core SNPs were identified by Mummer v3.2. ClonalFrameML was used to infer and then remove recombination, finally identifying 28,582 recombination-free core SNPs. The PROV023 isolate from C02.S03 served as the outgroup to determine the evolutionary relationships/distances for C01.S01, C01.S02, and C01.S05. The confidence value in the major branch nodes were over 95%. The isolates fully sequenced in this study were marked with black asterisks. The first two columns, from left to right, showed the isolated hospitals and the isolated dates. The plasmid-carrying isolates were marked with the blue block or the red block (corresponding to presence of *bla*_NDM-1_ or not respectively) in the remaining five columns. The local *bla*_NDM-1_ genetic environments were shown on the left side and the homologous regions were marked in shadow. The lines were drawn between the plasmids and the local *bla*_NDM-1_ genetic environments, representing which genetic environment was located in each indicated plasmid.

To further investigate plasmid-mediated dissemination of *bla*_NDM-1_ in *Providencia*, a maximum-likelihood phylogenetic tree (Figure 6) was constructed using a collection of 116 *Providencia* isolates, which harboured the plasmids (either carrying *bla*_NDM-1_ or not) of the above five Inc groups, from the above-described three dominant *Providencia* genospecies C01.S01 (10/116, 8.62%), C01.S02 (82/116, 70.69%), and C01.S05 (24/116, 20.69%). A total of 135 plasmids of the above five Inc groups were identified in these 116 isolates which were composed of 34 IncC plasmids, 38 Inc_pCHS4.1-3_ plasmids, 37 Inc_pPROV114-NR_ plasmids, 24 Inc_pPrY2001_ plasmids, and two IncW plasmids. The two IncW plasmids were identified in two clonal isolates with no obvious phylogenetic difference in C01.S05, while the 133 plasmids of IncC, Inc_pPROV114-NR_, Inc_pCHS4.1-3_, and Inc_pPrY2001_ were distributed in a wide range of clades on the phylogenetic tree (Figure 6). Compared to IncW plasmids, IncC, Inc_pPROV114-NR_, Inc_pCHS4.1-3_, and Inc_pPrY2001_ plasmids were more prevalent in *Providencia*, but randomly distributed among diverse lineages in different genospecies (Figure 6). The spread of these plasmids might be accidental evolutionary events and the plasmid-carrying *Providencia* isolates did not form dominant clades.

Nineteen (38%), five (10%), seven (14%), 18 (36%), and one (2%) of the above 34 IncC plasmids, 38 Inc_pCHS4.1-3_ plasmids, 37 Inc_pPROV114-NR_ plasmids, 24 Inc_pPrY2001_ plasmids, and two IncW plasmids carried *bla*_NDM-1_ genes, respectively; in total, quite a large portion (50/135, 37.04%) of these 135 plasmids had evolved to acquired *bla*_NDM-1_ genes. These 50 *bla*_NDM-1_-positive plasmids were discretely distributed among eight clades in C01.S02 (36/50, 72%), three clades in C01.S05 (11/50, 22%), and two clades in C01.S01 (3/50, 6%).

The local *bla*_NDM-1_ genetic environments within all the 50 *bla*_NDM-1_-carrying plasmids from the global 580 isolates could be divided into four distinct forms, namely intact Tn*125* [43–45] and three Tn*125* derivatives Tn*125-1*, Tn*125-2*, and Tn*125-3* (Figure 6). Tn*125* had a transposon structure of *bla*_NDM-1_-*ble*_MBL_-*trpF*-*dsbD*-*cutA*-*groEL*-*ISCR27* bracketed by two copies of ISA*ba125*. Tn*125-1* had undergone the partial deletions of ISA*ba125* at both boundaries. Tn*125-2* had the truncation of ISA*ba125* at the left boundary and that of *dsbD* at the right boundary, losing a large piece of sequence contacting *cutA*, *groES*, *groEL*, *tnpA*, and *ISCR27* that was associated with capturing *bla*_NDM-1_ [46]. Tn*125-3* was a short residual of Tn*125* and only contained *bla*_NDM-1_, *ble*_MEL_, and the *tnpA* and *trrpF* remnants.

By analysing the distribution of the same Inc group plasmids with the identical local *bla*_NDM-1_ genetic environment on the phylogenetic tree, we observed that IncC plasmids with Tn*125-3*, Inc_pPROV114-NR_ plasmids with Tn*125-3*, Inc_pPrY2001_ plasmids with Tn*125*, and Inc_pPrY2001_ plasmids with Tn*125-3* were contained in distinct lineages of C01.S01, C01.S02, and C01.S05, presenting the evidence of parallel horizontal transfer of various Tn*125* derivatives into existing *bla*_NDM-1_-empty plasmids belonging to multiple Inc groups. However, we did not observe such horizontal transfer events for Inc_pCHS4.1-3_ and IncW plasmids, which might be due to the lack of enough sequenced genomes analysed. Furthermore, we observed three distinct clonal expansion events of *Providencia* isolates with the combinations of C01.S02 + Inc_pCHS4.1-3 _+ Tn*125-2*, C01.S02 + IncC + Tn*125-3*, and C01.S05 + IncC + Tn*125-3* (Figure 6), respectively, indicating that vertical clonal expansions of *Providencia* isolates harbouring *bla*_NDM-1_-carrying plasmids drove the dissemination of *bla*_NDM-1_ genes. Taken together, *bla*_NDM-1_-positive plasmids of IncC, Inc_pPROV114-NR_, Inc_pPrY2001_, and Inc_pCHS4.1-3_ were highly involved in horizontal transfer or vertical clonal expansion events, majorly contributing to plasmid-mediated *bla*_NDM-1_ dissemination in *Providencia*.

## Discussion

The conventional species classification measure based on 16S rDNA sequences causes the frequent misclassification due to high similarity of 16S rDNA sequences among different *Providencia* species [4,11,47,48]. Although additional marker sequences such as housekeeping genes have been used to perform *Providencia* taxonomic assignment [47,49], the big problem is the insufficient genetic information for resolving the genetically diverse *Providencia* species. It is difficult to exactly assign a *Providencia* strain into the existing species even based on its genome sequence due to the comparatively longer distance to the conventional *Providencia* species [47]. In this study, a genome sequence-based species classification scheme for *Providencia* based on a collection of 580 global *Providencia* genomes was established to redefine the 11 conventional *Providencia* species into seven primary genocomplexes and 18 secondary genospecies. Compared to previous genome studies of *Providencia* [13,47], this study enrolled the largest *Providencia* genome dataset to establish the species classification scheme, which provided an extensively updated reference for *Providencia* species identification and the improved accuracy of genome sequence-driven species discrimination. The combined use of phylogenetic distances and pairwise ANI values across inner- and inter-species would provide a paradigm strategy for microbial species classification.

As shown in this study, *Providencia* isolates especially those from the three prevalent genospecies C01.S02, C01.S05, and C05.S01 carried diverse and abundant antimicrobial resistance genes and displayed high resistance ratios against multiple classes of drugs. The rapid development of MDR in *Providencia* might be primarily due to the extensive use and abuse of antibiotics [39,50]. It has been reported that there is a correlation between the increased colistin consumption and the increasing prevalence of drug resistant *Providencia* infections [39], and that the increased usage of colistin might have exerted significant pressure on the selection of *Providencia* in Romania [50]. *Providencia* might serve as the reservoir of resistance genes, enabling the transfer of resistance genes within *Providencia* and to other bacteria [51,52].

Our study identified a ratio of 14.14% (82/580) of *bla*_NDM-1_-positive *Providencia* isolates, indicating an emergence of challenge for clinical therapeutics caused by carbapenem resistance [18,21,53,54]. We further analysed the genetic basis of dissemination of *bla*_NDM-1_ in *Providencia*. On the one hand, we identified five Inc groups of *bla*_NDM-1_-carrying plasmids namely IncC [21], IncW [41,55], Inc_pCHS4.1-3_, Inc_pPROV114-NR_, and Inc_pPrY2001_, among which the last three were newly designated. IncC plasmids are mostly prevalent among *Providencia*, which is consistent with the previous study [21,22]. All these five Inc groups of *bla*_NDM-1_-carrying plasmids contain the complete conjugation regions, which would accelerate the spread of *bla*_NDM-1_ within *Providencia* and to other bacteria [21,37,40,56,57]. On the other hand, we observed the parallel horizontal transfer of local *bla*_NDM_ genetic environments (manifesting as intact Tn*125* and its various truncated derivatives) into the existing *bla*_NDM_-empty plasmids belonging to different Inc groups. In addition, our previous studies have reported the capture of local *bla*_NDM_ genetic environments by the accessory genetic elements embedded within *Providencia* chromosomes [42,45]. The exogenous integration of intact Tn*125* is mostly likely due to transposon jumps, while that of its truncated derivatives should be driven by homologous recombination [58].

In summary, we conducted a genomic epidemiology analysis on the 580 global sequenced *Providencia* isolates, including 257 ones sequenced in this study. Firstly, a genome sequence-based species classification scheme for *Providencia* was established, and the conventional 11 *Providencia* species were redefined into seven primary genocomplexes and 18 secondary genospecies, providing an extensively updated reference for *Providencia* species discrimination based on the largest *Providencia* genome dataset to date. Secondly, the prevalence of drug susceptibility and antimicrobial resistance genes were screened among all the genocomplexes/genospecies, disclosing the presence of diverse and abundant antimicrobial resistance genes and high resistance ratios against multiple classes of drugs in *Providencia*. Finally, the distribution of *bla*_NDM-1_-carrying plasmids and their local *bla*_NDM-1_ genetic environments was tracked among the three prevalent genospecies, revealing horizontal transfer or vertical clonal expansion of *bla*_NDM-1_ in *Providencia* driven by various Inc groups of plasmids. Data presented here would provide a comprehensive genomic view of species differentiation, antimicrobial resistance prevalence, and plasmid-mediated *bla*_NDM-1_ dissemination in *Providencia*.

## Supplementary Material

4_Table_S1_20230924

2_Supplementary_Data_R1_20230924
