# Supplementary material for: Genomic epidemiology and heterogeneity of Providencia and their blaNDM-1-carrying plasmids
Source: Emerg Microbes Infect. 2023 Nov 8;12(2):2275596. doi: 10.1080/22221751.2023.2275596 (PMC10796120; doi:10.1080/22221751.2023.2275596)
Supplement: 2_Supplementary_Data_R1_20230924 [file TEMI_A_2275596_SM5708.pdf]

Supplementary Data

Genomic epidemiology and heterogeneity of *Providencia* and their *bla*$_{NDM-1}$-carrying plasmids

Peng Wang[a#], Cuidan Li[b#], Zhe Yin[a], Xiaoyuan Jiang[b], Xinyue Li[a], Xiaofei Mu[a], Nier Wu[a], Fei Chen[b,c]*, and Dongsheng Zhou[a]*

This file includes Table S2, and Figure S1 to Figure S7. Table S1 is presented as a separate document.

(1) Table S1. Information of our 257 Chinese *Providencia* isolates
(2) Table S2. Five Inc groups of *bla*$_{NTM}$-carrying plasmids among the 580 global *Providencia* isolates
(3) Figure S1. Bacteria collection in this study
(4) Figure S2. Distribution of antimicrobial resistance genes in the 580 global *Providencia* isolates
(5) Figure S3. Comparison of antimicrobial resistance genes between our 257 Chinese *Providencia* isolates and additional 323 *Providencia* isolates available in GenBank in different genospecies
(6) Figure S4. Drug susceptibility testing data of our 257 Chinese *Providencia* isolates
(7) Figure S5. Antimicrobial resistance profiles of our 257 Chinese *Providencia* isolates among the genospecies
(8) Figure S6. Alignment of the five Inc groups of plasmids from the 580 global *Providencia* isolates
(9) Figure S7. Correlation heatmap of antimicrobial resistance with resistance genes

Table S2. Five Inc groups of *bla*$_{NDM-1}$-carrying plasmids among the 580 global *Providencia* isolates

Inc group	*bla*$_{NDM-1}$ local genetic environment	Total (n = 580)			This study (n = 257)			GenBank (n = 323)		
		Number of plasmids	Percent	Rank	Number of plasmids	Percent	Rank	Number of plasmids	Percent	Rank
IncC	-	43	7.41%		14	5.44%		29	8.97%	
	ΔTn*125-2*	7	1.20%	**Top3**	5	1.94%	**Top2**	2	0.61%	
	ΔTn*125-3*	28	4.82%	**Top1**	11	4.28%	**Top1**	17	5.26%	**Top1**
Inc$_{pPROV114-NR}$	-	38	6.55%		34	13.22%		4	1.23%	
	ΔTn*125-1*	1	0.17%		-	-		1	0.30%	
	ΔTn*125-2*	4	0.68%		4	1.55%		-	-	
Inc$_{pCHS4.1-3}$	-	41	7.06%		35	13.61%		6	1.85%	**Top3**
	ΔTn*125-1*	1	0.17%		-	-		1	0.30%	
	ΔTn*125-2*	4	0.68%		4	1.55%	**Top3**	-	1.23%	
	ΔTn*125-3*	5	0.86%		2	0.77%		3	0.92%	
Inc$_{pPrY2001}$	-	7	1.20%		5	1.94%		2	0.61%	
	Tn*125*	5	0.86%		4	1.55%	**Top3**	1	0.30%	
	ΔTn*125-1*	4	0.68%		-	-		4	1.23%	
	ΔTn*125-3*	9	1.55%	**Top2**	-	-		9	2.78%	**Top2**
IncW	-	1	0.17%		1	0.38%		-	-	
	ΔTn*125-3*	1	0.17%		1	0.38%		-	-	
Total		199	34.3%		120	20.68%		79	13.62	

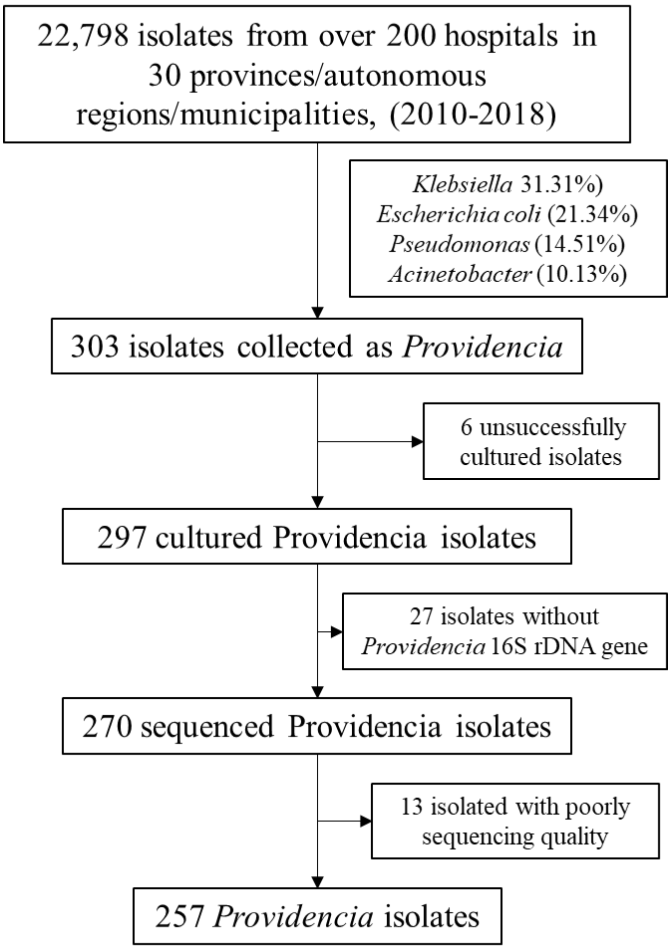

Figure S1. Bacteria collection of 257 *Providencia* isolates

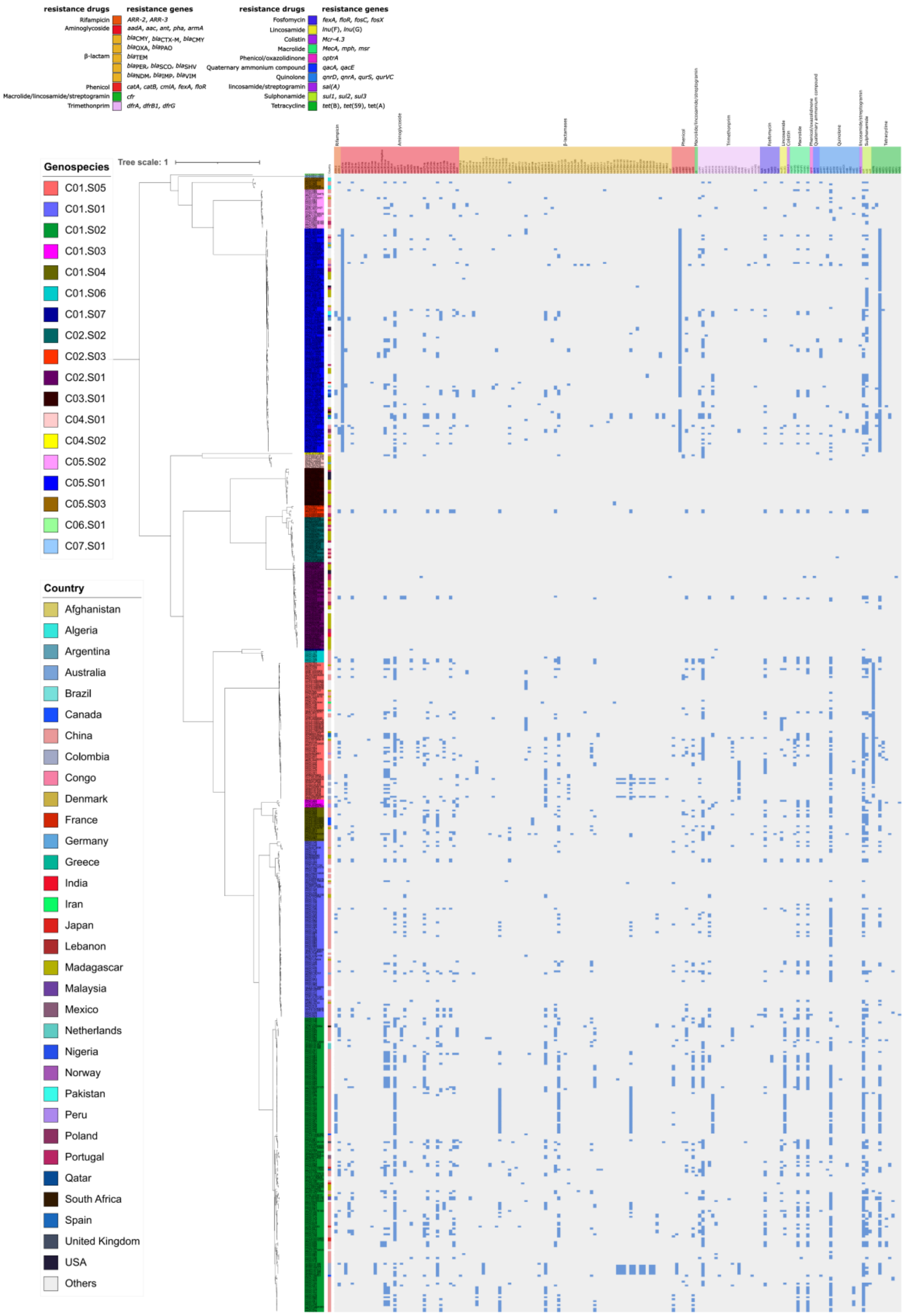

Figure S2. Distribution of antimicrobial resistance genes in the 580 global *Providencia* isolates. The phylogenetic tree was constructed using the 13,499 core SNPs from the 580 global *Providencia* genomes. The confidence level for the major branch nodes was over 95%. The first column mapped on the tree represented the

genospecies for the isolates, and the second column indicated the countries of the isolates. The following matrix showed the presence of resistance genes indicated by blue coloured cells. The top of the matrix indicated the names of resistance genes, which were coloured and grouped based on their categories.

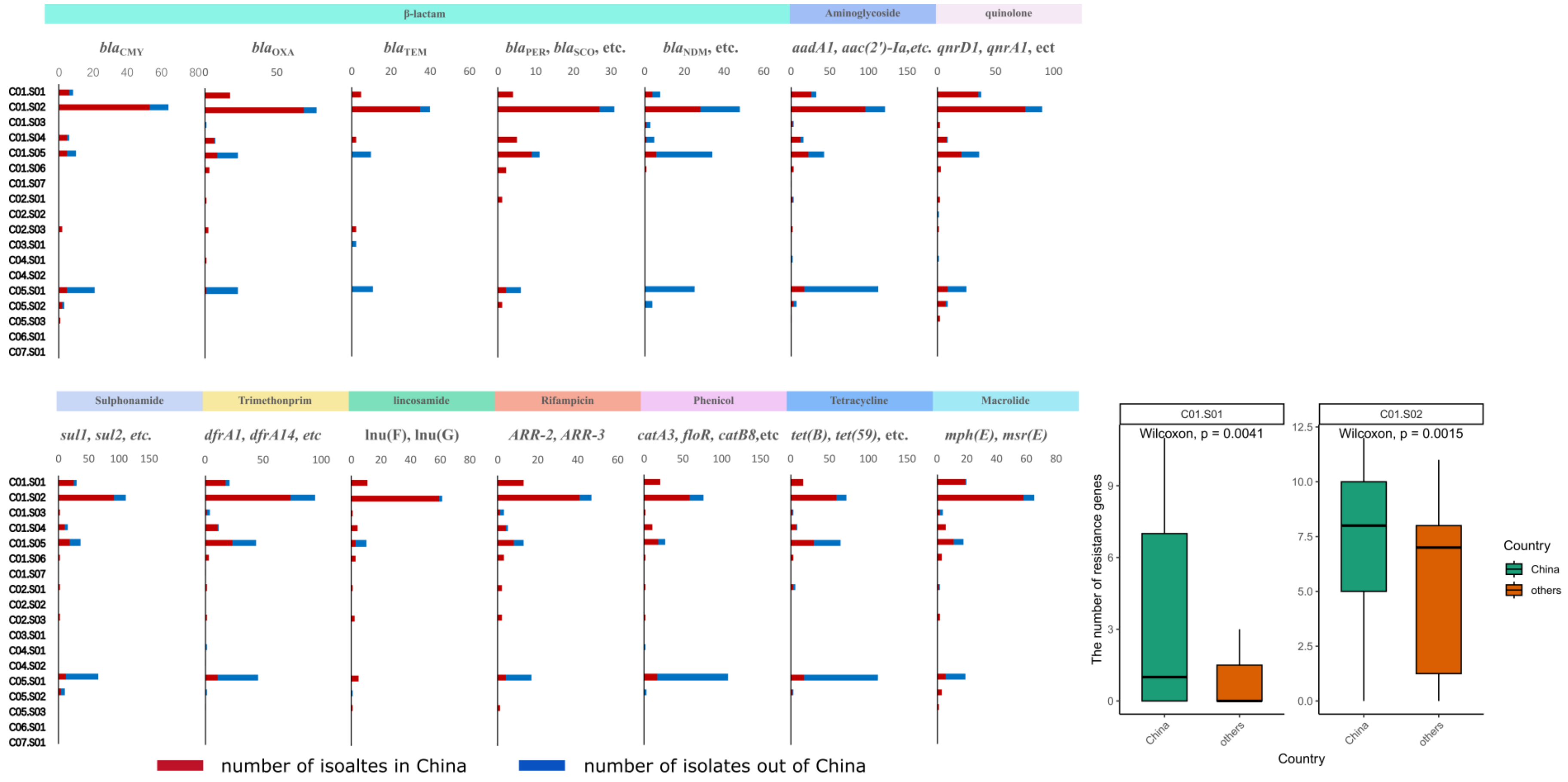

Figure S3. Comparison of antimicrobial resistance genes between our 257 Chinese *Providencia* isolates and additional 323 *Providencia* isolates available in GenBank in different genospecies. The red and blue bars represented our 257 Chinese *Providencia* isolates and additional

323 *Providencia* isolates available in GenBank, respectively. (**b**). Comparison of resistance genes between Chian and other countries in C01.S01 and C01.02.

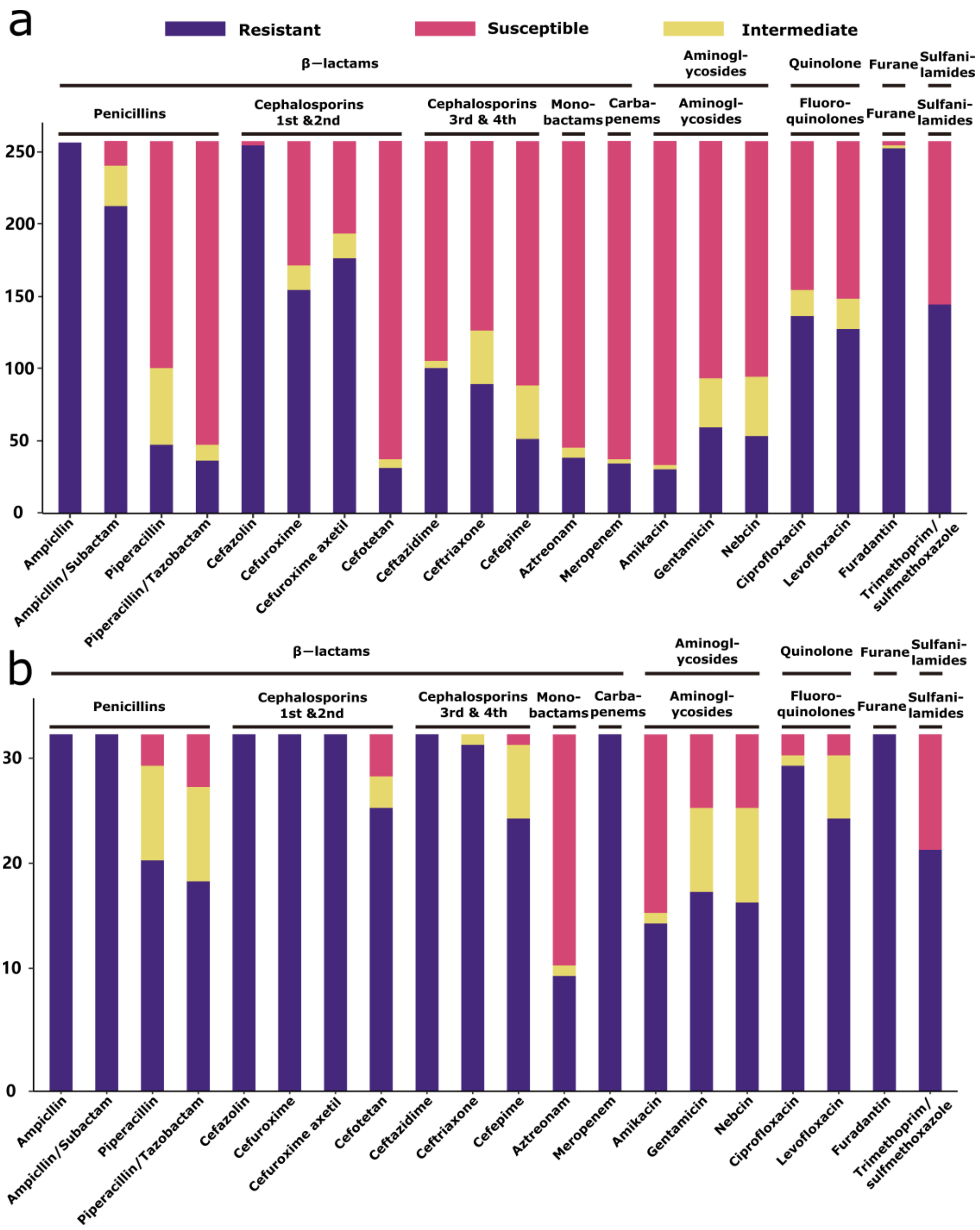

Figure S4. Drug susceptibility testing data of our 257 Chinese *Providencia* isolates. Antimicrobial resistance profiles of all the 257 isolates (**a**) and those of the 33 *bla*$_{NDM-1}$-carrying isolates from these 257 isolates (**b**). A total of 20 antibiotics in nine classes were tested.

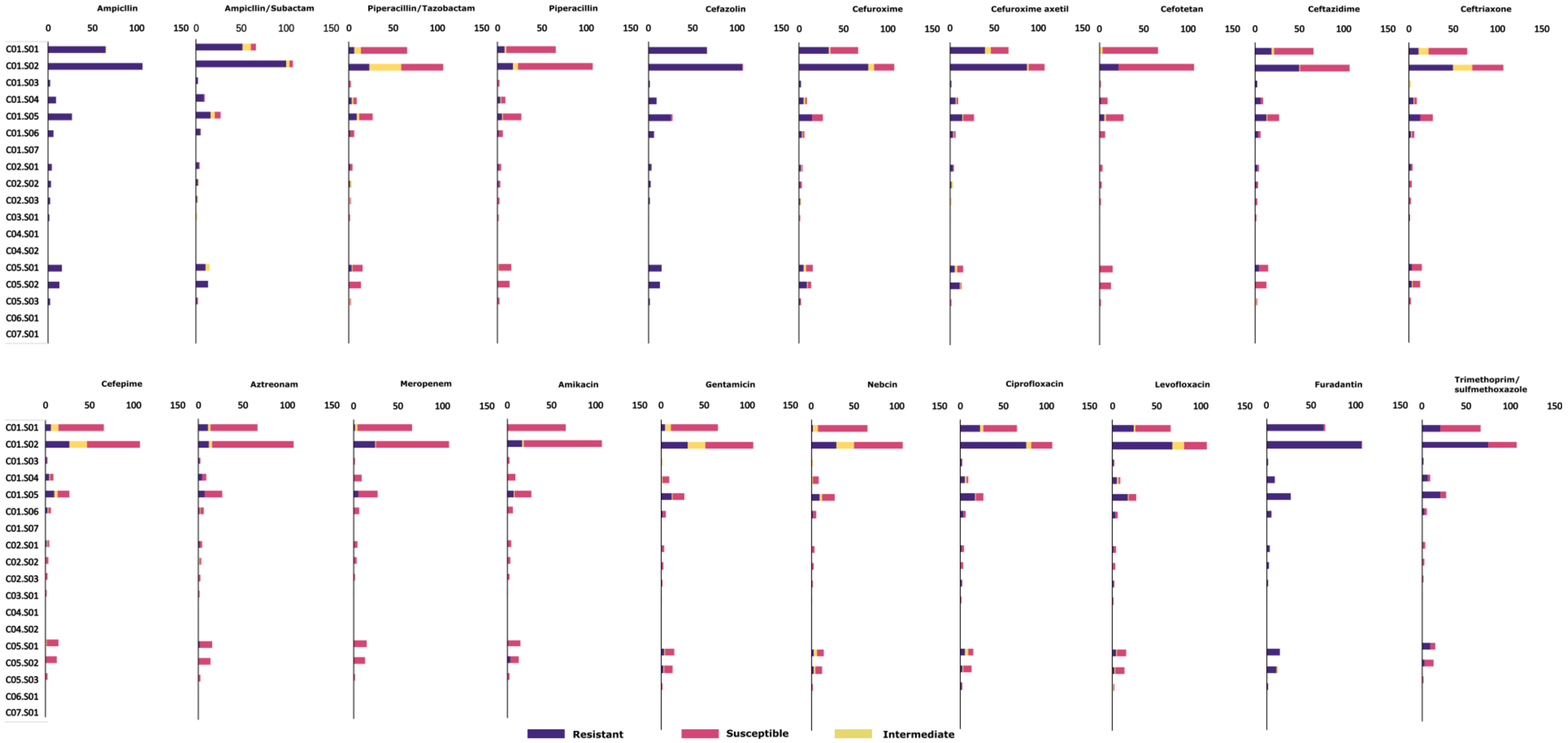

Figure S5. Antimicrobial resistance profiles of our 257 Chinese *Providencia* isolates among the genospecies.

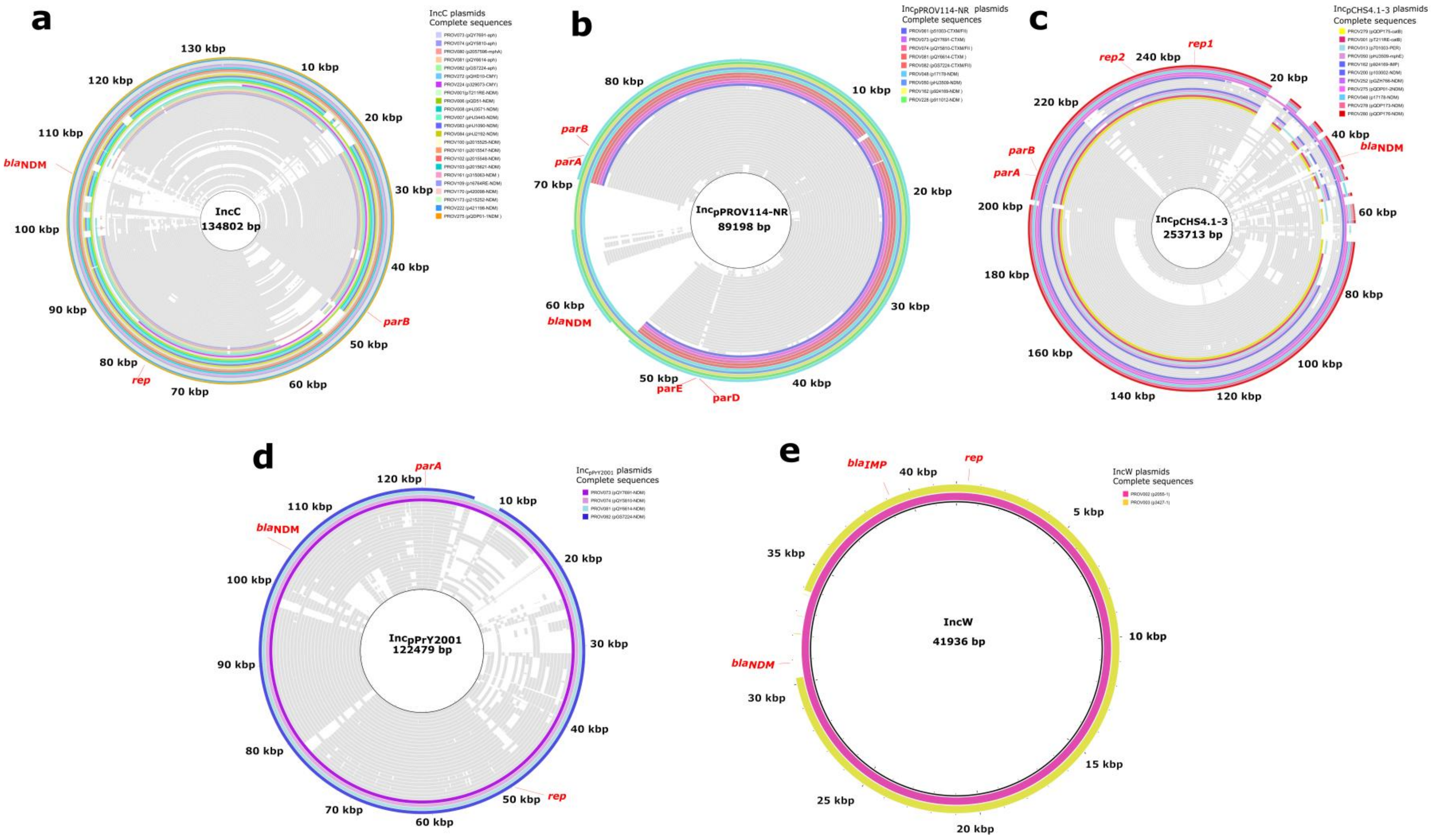

Figure S6. Alignment of the five Inc groups of plasmids from the 580 global *Providencia* isolates. The color rings represented the fully sequenced plasmids, while the grey rings stood for those with draft sequences. (**a**) Alignment of 79 IncC plasmids including 36 *bla*$_{NDM-1}$-carrying

plasmids. (**b**) Alignment of 43 Inc$_{\text{pPROV114-NR}}$ plasmids including five *bla*$_{\text{NDM-1}}$-carrying plasmids. (**c**) Alignment of 51 Inc$_{\text{pCHS4.1-3}}$ plasmids including 10 *bla*$_{\text{NDM-1}}$-carrying plasmids. (**d**) Alignment of 29 Inc$_{\text{pPrY2001}}$ plasmids including 20 *bla*$_{\text{NDM-1}}$-carrying plasmids. (**e**) Alignment of two IncW plasmids including one *bla*$_{\text{NDM-1}}$-carrying plasmid.

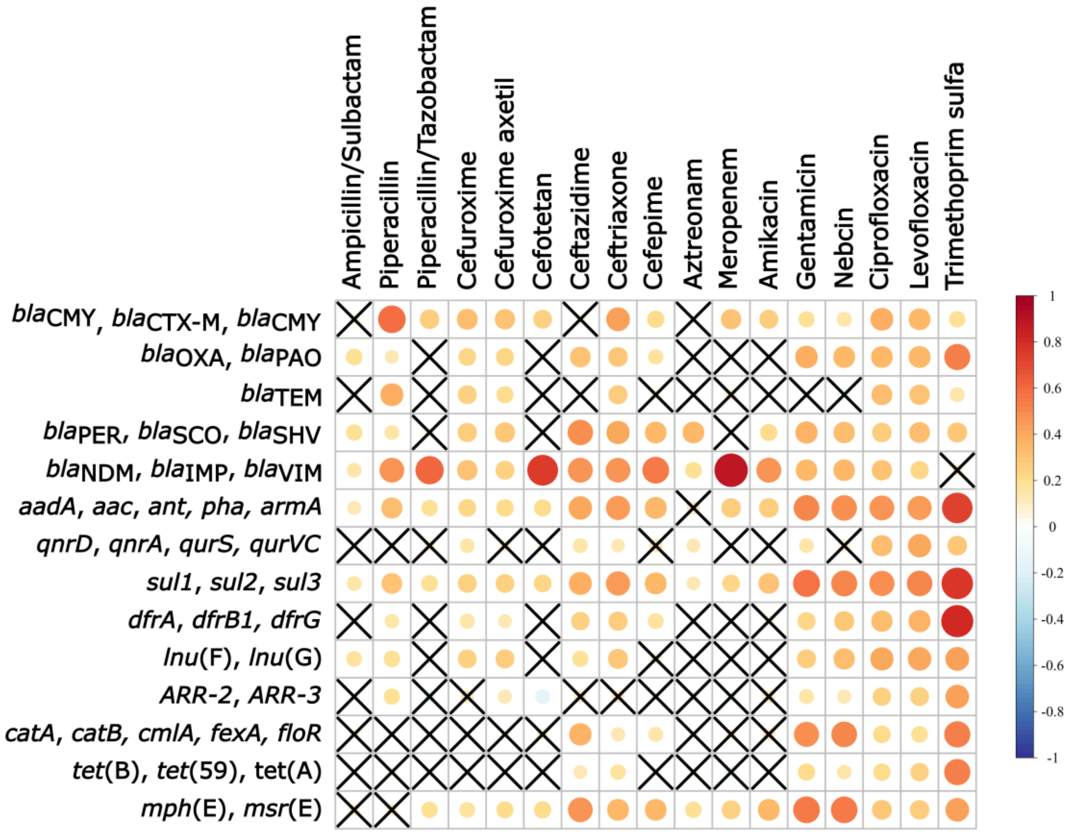

Figure S7. Correlation heatmap of antimicrobial resistance with resistance genes.
